# It’s more than low BMI: prevalence of cachexia and associated mortality in COPD

**DOI:** 10.1186/s12931-019-1073-3

**Published:** 2019-05-22

**Authors:** Merry-Lynn N. McDonald, Emiel F. M. Wouters, Erica Rutten, Richard Casaburi, Stephen I. Rennard, David A. Lomas, Marcas Bamman, Bartolome Celli, Alvar Agusti, Ruth Tal-Singer, Craig P. Hersh, Mark Dransfield, Edwin K. Silverman

**Affiliations:** 1 Division of Pulmonary, Allergy and Critical Care Medicine, University of Alabama at Birmingham, Birmingham, AL USA; 2 Department of Genetics, University of Alabama at Birmingham, Birmingham, AL USA; 3 Centre of expertise for chronic organ failure, Horn, the Netherlands and Department of Respiratory Medicine, Maastricht University Medical Center, Maastricht, the Netherlands; 4 Rehabilitation Clinical Trials Center, Los Angeles Biomedical Research Institute at Harbor Harbor-UCLA Medical Center, Torrance, CA USA; 5 Department of Medicine, Nebraska Medical Center, Omaha, NE USA; 6 Biopharma R&D, AstraZeneca, Cambridge, UK; 7 UCL Respiratory, University College London, London, UK; 8 Center for Exercise Medicine and Departments of Cell, Developmental & Integrative Biology; Medicine; and Neurology, University of Alabama at Birmingham, Birmingham, AL USA; 9 Division of Pulmonary and Critical Care, Brigham and Women’s Hospital, Boston, MA USA; 10 Fundació Investigació Sanitària Illes Balears (FISIB), Ciber Enfermedades Respiratorias (CIBERES), Barcelona, Catalunya Spain; 11 Thorax Institute, Hospital Clinic, IDIBAPS, Univ. Barcelona, Barcelona, Spain; 12 GSK R&D, Collegeville, PA USA; 13 Channing Division of Network Medicine, Brigham and Women’s Hospital, Boston, MA USA; 14 Lung Health Center, University of Alabama at Birmingham, 701 19th Street S, LHRB 440, Birmingham, AL 35233 USA; 15 Center for Exercise Medicine, University of Alabama at Birmingham, 701 19th Street S, LHRB 440, Birmingham, AL 35233 USA

**Keywords:** COPD, Cachexia, BODE, Weight loss, BMI

## Abstract

**Background:**

Cachexia is associated with increased mortality risk among chronic obstructive pulmonary disease (COPD) patients. However, low body mass index (BMI) as opposed to cachexia is often used, particularly when calculating the BODE (BMI, Obstruction, Dyspnea and Exercise) index. For this reason, we examined mortality using a consensus definition and a weight-loss definition of cachexia among COPD cases and compared two new COPD severity indices with BODE.

**Methods:**

In the current report, the consensus definition for cachexia incorporated weight-loss > 5% in 12-months or low BMI in addition to 3/5 of decreased muscle strength, fatigue, anorexia, low FFMI and inflammation. The weight-loss definition incorporated weight-loss > 5% or weight-loss > 2% (if low BMI) in 12-months. The low BMI component in BODE was replaced with the consensus definition to create the CODE (Consensus cachexia, Obstruction, Dyspnea and Exercise) index and the weight-loss definition to create the WODE (Weight loss, Obstruction, Dyspnea and Exercise) index. Mortality was assessed using Kaplan-Meier survival and Cox Regression. Performance of models was compared using C-statistics.

**Results:**

Among 1483 COPD cases, the prevalences of cachexia by the consensus and weight-loss definitions were 4.7 and 10.4%, respectively. Cachectic patients had a greater than three-fold increased mortality by either the consensus or the weight-loss definition of cachexia independent of BMI and lung function. The CODE index predicted mortality slightly more accurately than the BODE and WODE indices.

**Conclusions:**

Cachexia is associated with increased mortality among COPD patients. Monitoring cachexia using weight-loss criteria is relatively simple and predictive of mortality among COPD cases who may be missed if only low BMI is used.

**Electronic supplementary material:**

The online version of this article (10.1186/s12931-019-1073-3) contains supplementary material, which is available to authorized users.

## Background

Cachexia is characterized by a rapid weight-loss, primarily caused by loss of muscle with or without loss of fat, in individuals suffering from a chronic illness [1]. Though the precise definition of cachexia is controversial, it is generally agreed that cachexia is associated with a greatly increased risk of mortality among COPD cases [2, 3]. By general population prevalence, it has been estimated that there are 1.4 times as many cases with cachexia in COPD compared to cancer cases [4]. Important systemic manifestations of COPD may be captured using the well-established BODE (BMI, Obstruction, Dyspnea and Exercise) index, which ranges from 0 to 10 [5, 6]. COPD patients with higher BODE scores have a higher risk of death and the index outperforms the mortality prediction of its individual components which includes low BMI (BMI < 21) [5]. However, patients across the entire BMI spectrum may be at risk of developing cachexia [7, 8]. Evaluating COPD cachexia using only BMI may underdiagnose overweight patients as it may mask significant muscle wasting [7, 8].

The current consensus definition for cachexia diagnosis among COPD, heart failure, and cancer cases incorporates weight-loss > 5% in the last 12 months in addition to three out of five criteria: 1) decreased muscle strength; 2) fatigue; 3) anorexia; 4) low fat-free mass index (FFMI); and 5) evidence of increased inflammatory markers (C-reactive protein (CRP), interleukin-6 (IL6), etc.), anemia or low serum albumin [1]. A challenge for studying cachexia in COPD is that most often all of the criteria for the consensus definition are not available as part of routine clinical care nor in large epidemiologic cohorts. Recently, cachexia was characterized among cancer cases using simpler criteria (weight-loss > 5% or, if low BMI, weight-loss > 2%) than the consensus definition [9].

As the current estimates of COPD cachexia have been largely based on low BMI, we aimed to estimate the prevalence of cachexia and its associated mortality in a large cohort of COPD cases followed for three years in the ECLIPSE study using two sets of cachexia criteria that we termed consensus and weight-loss (Table 1). The consensus criteria used in the current analyses differed from the standard consensus definition of cachexia in that information was not available to assess muscle strength or all components of abnormal biochemistry [1]. Low 6-min walk distance (6MWD) was used as a surrogate for reduced muscle strength. We further aimed to examine the prevalence of cachexia stratified by severity of COPD and BMI category. We hypothesized that COPD cases with cachexia would have an increased risk of mortality. We further hypothesized that cachexia would be more common among more severe COPD cases as defined by GOLD stage but would be prevalent across all categories of BMI (low to obese). We also replaced the low BMI component of BODE with the criteria for consensus and weight-loss cachexia to create the CODE (Cachexia, Obstruction, Dyspnea and Exercise) and WODE (Weight loss, Obstruction, Dyspnea and Exercise) indices and compared performance in terms of mortality prediction.

**Table 1:** Criteria used to define cachexia by either consensus or weight-loss definitions among COPD cases in ECLIPSE

Definition	Criteria
*Consensus*	Weight-loss > 5% in 12-months or low BMI in addition to 3 out of 5 of decreased muscle strength, fatigue, anorexia, FFMI and abnormal biochemistry (hemoglobin 12 g/dL or CRP > 5 mg/L)
*Weight-loss*	Weight-loss > 5%, or, in the presence of low BMI, weight-loss > 2%

*FFMI* fat-free mass index, *CRP* C-reactive protein, *BMI* body mass index

## Methods

### Study population

The ECLIPSE (Evaluation of COPD Longitudinally to Identify Predictive Surrogate Endpoints) datasets analyzed during the current study are available in the database of genotypes and phenotypes (dbGaP) as phs001252.v1.p1 (https://www.ncbi.nlm.nih.gov/projects/gap/cgi-bin/study.cgi?study_id=phs001252.v1.p1). All analyses were performed after obtaining approval from the Institutional Review Board of the University of Alabama at Birmingham. ECLIPSE (SCO104960, NCT00292552, www.eclipse-copd.com), which has been described in detail elsewhere [10], was a non-interventional, observational, multicenter study which followed COPD patients and controls that were either nonsmokers or who had a smoking history of ≥10 pack-years, aged 45–75 years from 46 centers across 12 countries over 3 years with 8 visits.

In total,1825 ECLIPSE participants with COPD at both baseline and Year 1 met consent criteria for the current analyses (Additional file 1: Figure S1). At Year 1, 1483 COPD cases were assessed for the consensus definition of cachexia, and weight-loss associated with cachexia (Additional file 1: Figure S1). Only COPD cases with data available to characterize both cachexia traits were included in the current analyses. COPD was diagnosed using standardized post-bronchodilator spirometry at both baseline and Year 1 of the study. Percent weight-loss was calculated by subtracting the Year 1 weight from baseline weight at study entry, dividing by the baseline weight and multiplying by 100. Participants who exhibited weight-loss exceeding threshold values (weight-loss more than 5% or if BMI < 20 kg/m^2^ and weight-loss more than 2%) at Year 1 with evidence that weight was regained at a later visit between Years 1 and 3 in the study were coded as not having weight-loss. FFMI was measured using bioelectrical impedance. Low FFMI was classified if FFMI was below the 5th percentile value in age, BMI and sex-stratified data from healthy individuals in the UK Biobank [11]. Low BMI was defined as BMI < 20 kg/m^2^. 6MWD less than 350 m [12] was used as a surrogate for reduced muscle strength (see Discussion). Fatigue was classified by Functional Assessment of Chronic Illness Therapy (FACIT) score less than 34 [13]. Anorexia was classified if a participant reported they did not feel like eating in the last week for 3 or more days. Abnormal biochemistry was defined as CRP > 5 mg/L or anemia (hemoglobin < 12 g/dL). All variables used for coding the cachexia traits were collected at the Year 1 visit, with the exception of fatigue and anorexia, which were only collected at baseline. Two new indexes, the WODE and CODE, were coded by replacing the BMI component of BODE with weight-loss cachexia or consensus cachexia, respectively.

### Statistical analysis

All analyses were performed using the statistical software R. The chi-square test of homogeneity was used to test for differences between categorical variables. The two-sided student’s t-test was used to test for differences between continuous variables. The Cochran Armitage test for trend was used to assess trends in the prevalence of cachexia and weight-loss with categorical variables. Survival modeling was performed using Kaplan-Meier curves and Cox proportional hazards regression. Cox proportional hazards models included adjustment for continuous variables age and smoking duration (pack-years) and categorical variables sex and BMI category (low < 18.5 kg/m^2^, normal between 18.5 kg/m^2^ and 25 kg/m^2^, overweight between 25 kg/m^2^ and 30 kg/m^2^ and obese > 30 kg/m^2^). BMI category was classified based on Year 1 BMI even in those who changed BMI category during the mortality follow-up. Mortality prediction accuracy of each of three indices (BODE, CODE and WODE) was evaluated by calculating C-statistics.

## Results

### Study characteristics

A total of 1483 COPD cases from the ECLIPSE study with complete information available to classify consensus and weight-loss cachexia (Table 1) were included in the analysis. Among the COPD cases analyzed, there were more men (65.3%) than women. The median age was 64 years and the median BMI was 25.9 (Table 2). COPD cases had median smoking history of 44 pack-years and were distributed in GOLD spirometry grades 2 through 4, which were previously referred to as “GOLD stages” (Table 2). COPD cases included in our analyses differed from those excluded due to missing information for coding consensus or weight-loss cachexia with respect to age and severity of COPD defined by FEV1% predicted and GOLD stage (Table 2). COPD cases excluded from analyses were on average older and comprised of more severe cases with respect to lung function.

**Table 2:** Characteristics of ECLIPSE Study COPD cases included and excluded from analyses

Descriptive	COPD Cases Included	COPD Cases Excluded	*P*-value
N	1483	342	
Sex (% Male)	65.3	67	0.6
Age	64 (10)	66 (11)	**0.04**
BMI	25.9 (7.1)	25.6 (6.2)	0.1
Current Smoking (%)	44 (29)	44 (26)	0.9
FEV1 (% predicted)	47.5 (23.5)	44.2 (28.9)	**0.02**
GOLD 2 (%)	44.9	38	**0.04**
GOLD 3 (%)	41.1	44.2	
GOLD 4 (%)	14	17.8	

*N* total number, *IQR* interquartile range, *BMI* body mass index

Notes: The chi-square test of homogeneity was used to test for differences between categorical variables. The two-sided student’s t-test was used to test for differences between continuous variables. All variables were measured at Year 1 Visit, with the exception of pack-years, which was based on the baseline visit

COPD cases were excluded if missing information necessary to code the consensus or weight-loss cachexia traits. Continuous variables (age, BMI, FEV1% predicted) are represented by median (IQR). Significant *p*-values are bolded

### Prevalence of cachexia and weight-loss among COPD cases

**Fig. 1:**
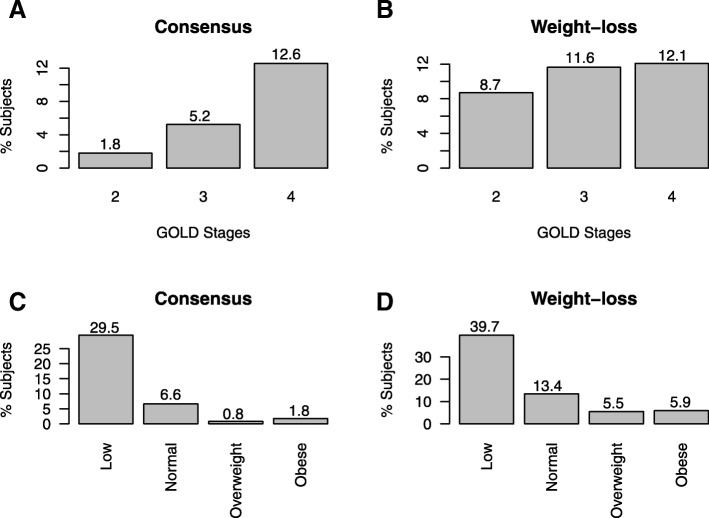
Prevalence of cachexia (consensus and weight-loss definitions) stratified by GOLD and BMI category among COPD cases at Year 1 Visit in ECLIPSE stratified by GOLD (**a**, **b**) and BMI category (**c**, **d**). Numbers above bars represent prevalence in each group. Percentage of COPD cases with cachexia by the consensus definition significantly (*p* < 0.001) increased with increasing GOLD stage, COPD cases with low BMI were more likely (*p* < 0.001) to have cachexia by both the consensus and weight-loss definitions. Note: Cochran Armitage test was used to assess trends

Among COPD cases, the prevalence of cachexia using the consensus definition was 4.7% and using the weight-loss definition was 10.4%. When the results were stratified by GOLD stage, the percentage of COPD cases with cachexia by the consensus definition significantly (*p* < 0.001) increased with increasing GOLD stage (Fig. 1a). Similarly, the percentage of COPD cases with cachexia by the weight-loss definition significantly (*p* < 0.05) increased with increasing GOLD stage (Fig. 1b). When the results were stratified by BMI category (normal, overweight, obese and low), COPD cases with low BMI were more likely (*p* < 0.001) to have cachexia by both the consensus and weight-loss definitions (Fig. 1c and d). However, there were cachectic COPD cases as defined by the consensus and weight-loss definitions among all categories of BMI. Participants with cachexia and weight-loss had significantly (*p* < 0.001) more emphysema at baseline and Year 1 (Additional file 2: Figure S2). There was no significant change in emphysema over Year 1 among COPD participants with cachexia and weight-loss (Additional file 3: Figure S3).

### Overlap in classification and relationship with mortality of cachexia among COPD cases

**Fig. 2:**
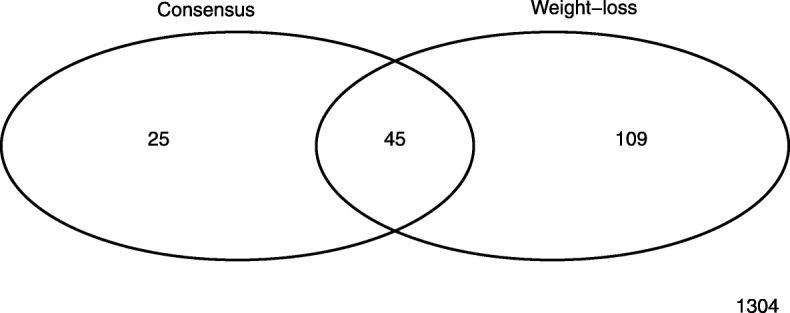
Overlap in classification of consensus and weight-loss classifications among COPD cases in ECLIPSE

**Fig. 3:**
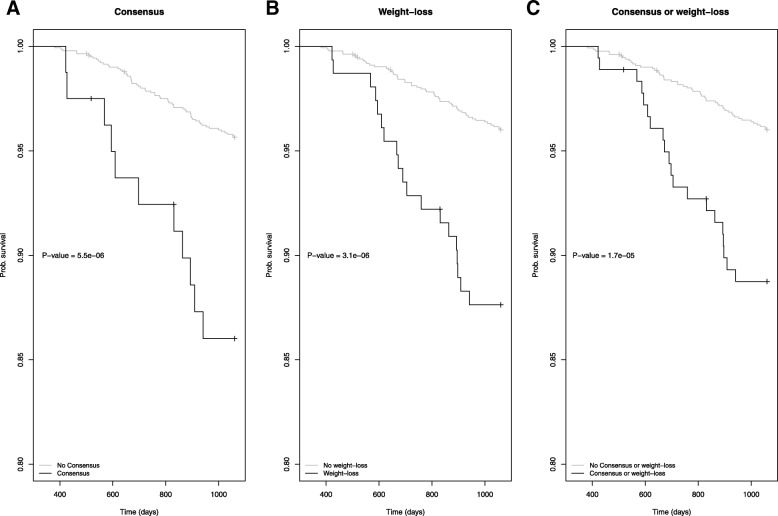
Kaplan Meier Survival Stratified by Cachexia as compared to the remaining cohort. **a**. Cachexia defined using consensus definition, **b**. Cachexia defined by weight-loss and **c**. Cachexia defined as either consensus definition or weight-loss

A total of 12.1% of COPD cases met the criteria for cachexia by the consensus or weight-loss definitions (Fig. 2). A total of 3.0% of COPD cases were classified as cachectic by both the consensus and weight-loss definitions of cachexia. In our analysis using longitudinal follow-up data, there were 72 deaths. The median follow-up time for the cohort was 2.9 years. COPD cases with cachexia by the consensus or weight-loss definition were more likely to die than those without cachexia (Fig. 3a, b, c). We further examined the relationships between cachexia and all-cause mortality using Cox regression modeling accounting for pack-years of smoking, BMI category, sex and age. When controlling for these additional covariates, cachexia by the consensus definition was still associated with an increased risk of death (Table 3 Consensus: Model Hazard Ratio (HR): 3.2, 95% CI 1.6–6.6, *p* = 0.001). This observation also held when cachexia was defined using the weight-loss definition (Table 3: Weight-Loss Model HR: 3.2 95% CI: 1.8–5.6, *p* < 0.001). Similarly, COPD subjects with either cachexia by the consensus definition or weight-loss were at an increased risk of death (Table 3: Consensus or Weight-Loss Model HR: 2.9, 95% CI 1.7–5.1, *p* < 0.001). In the Cox Proportional models, we also observed a significant reduced risk of death for overweight COPD patients in comparison with normal weight COPD patients (Consensus Model Overweight HR: 0.30, 95% CI 0.14–0.65, *p* = 0.002, Weight-Loss Model Overweight HR: 0.30, 95% CI 0.14–0.66, *p* = 0.003, Consensus or weight-loss Model Overweight HR: 0.31, 95% CI 0.14–0.68, p = 0.003). We contrasted the descriptives of overweight and normal BMI COPD participants in Additional file 4: Table S1. Overweight participants with COPD had better lung function defined by FEV1 percent predicted and fewer severe cases based on GOLD. However, all GOLD stages were represented among the overweight COPD cases. When COPD cases with weight-loss between baseline and Year 1 regained the weight at a later visit were coded as meeting the criteria for weight-loss, the increased risk of death associated with the consensus and weight-loss definitions remained significant (Additional file 5: Table S2: Consensus Model HR: 2.8 95% CI 1.4–5.7, *p* = 0.004, Weight-Loss Model: HR: 2.4 95% CI: 1.4–4.2, p = 0.002, Consensus or weight-loss Model HR: 2.3, 95% CI 1.3–4.0, *P* = 0.004) as the significant relationship with decreased risk of death associated with being overweight.

**Table 3:** Relationships between risk of death from any cause with consensus and weight-loss definitions of cachexia in COPD cases from ECLIPSE

Model	Covariate		HR (95% CI)	*P*-value
Model 1	Consensus		3.2 (1.6–6.6)	**0.001**
	Pack-years		1.0 (0.99–1.01)	0.1
	BMI category (ref = Normal)	Low	0.67 (0.23–2.0)	0.46
		Overweight	0.30 (0.14–0.65)	**0.002**
		Obese	1.5 (0.86–2.5)	0.16
	Sex (ref = males)		0.86 (0.49–1.5)	0.49
	Age		1.1 (1.0–1.1)	**< 0.001**
	FEV1% pred		0.98 (0.96–0.99)	**< 0.001**
Model 2	Weight-loss		3.2 (1.8–5.6)	**< 0.001**
	Pack-years		1.0 (0.998–1.01)	0.17
	BMI category (ref = Normal)	Low	0.68 (0.24–2.0)	0.48
		Overweight	0.30 (0.14–0.66)	**0.003**
		Obese	1.5 (0.89–2.7)	0.12
	Sex (ref = males)		0.78 (0.45–1.4)	0.39
	Age		1.1 (1.0–1.1)	**< 0.001**
	FEV1% pred		0.97 (0.96–0.99)	**0.003**
Model 3	Consensus or weight-loss		2.9 (1.7–5.1)	**< 0.001**
	Pack-years		1.0 (0.998–1.01)	0.16
	BMI category (ref = Normal)	Low	0.62 (0.21–1.8)	0.39
		Overweight	0.31 (0.14–0.68)	**0.003**
		Obese	1.6 (0.91–2.7)	0.11
	Sex (ref = males)		0.80 (0.46–1.4)	0.44
	Age		1.07 (1.03–1.1)	**< 0.001**
	FEV1% pred		0.97 (0.96–0.99)	**0.003**

FEV1% pred: forced expiratory volume in 1 s percent predicted, BMI: body mass index

Significant *p*-values are bolded

### Comparison of WODE and CODE indices with BODE

**Fig. 4:**
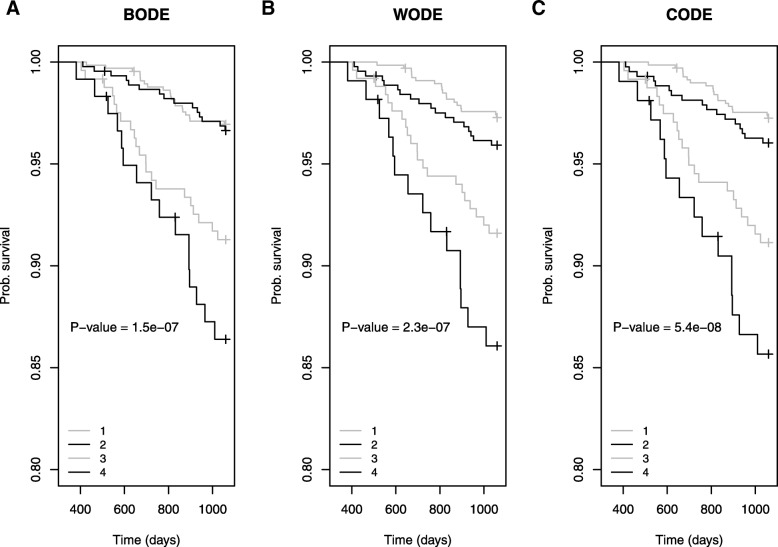
Kaplan Meier Survival Comparison between. **a**. BODE **b**. WODE and **c**. CODE quartiles

We coded two new indices, WODE and CODE by replacing the BMI component of BODE with either cachexia defined by weight-loss or the consensus definition. COPD cases with higher CODE scores were more likely to die than those with higher WODE or BODE scores. In the ECLIPSE cohort, we also observed a more uniform increase in risk of death for each increasing quartile for CODE and WODE scores in comparison with BODE (Fig. 4). We further assessed the precision of each metric by comparing survival times and probability of death using C-statistics. The C-statistics were comparable between all 3 indices (C_BODE_ = 0.72, C_WODE_ = 0.71, C_CODE_ = 0.73). Further, the *p*-value from the Kaplan-Meier survival based on CODE quartiles was also slightly more significant than for BODE and WODE quartiles (Fig. 4).

## Discussion

In the current investigation, the prevalence of cachexia in COPD cases ranges from 4.7 to 10.4% depending on the criteria used to define cachexia. Further, COPD cases representative of the entire BMI spectrum, not just those with low BMI, met the criteria for cachexia. Using either consensus definition or a simpler definition based on recent weight-loss, cachectic COPD cases were at an approximately three-fold increased risk of death. This increased risk of death was independent of BMI. These findings differ from other studies indicating increased risk of mortality for COPD patients with low BMI because patients across all BMI categories had cachexia. This underscores the importance of using more than low BMI to monitor patients for cachexia. Although the weight-loss definition of cachexia does not incorporate several key components of the consensus definition, it is comparatively simple to assess and highly predictive of patient survival. When we replaced the BMI component of BODE with either cachexia defined by weight-loss (WODE) or the consensus (CODE) definitions, CODE classified patients at risk of death slightly better than WODE or BODE; however, the results were comparable for all three indices. Our study has several strengths including recruitment of a large sample size of COPD patients with detailed phenotyping relevant to COPD and cachexia. These strengths were complemented by a relatively long follow-up period to examine survival and the impact of regaining weight.

We report the first study to apply a consensus definition of cachexia to a population of COPD cases to estimate prevalence. In the current investigation, the consensus and weight-loss definitions of cachexia demonstrated the prevalence ranged from 4.7 to 10.4%. With respect to cachexia defined by weight-loss, Barker et al. 2014 observed 15% of COPD patients exhibited a weight-loss > 5% in 16 months of follow-up which is similar to the rate observed in the current investigation [14]. Whereas, the prevalence of weight-loss was higher, 49%, in a cohort of COPD patients admitted to a pulmonary rehabilitation center [15]. Multiple sets of criteria of varying complexity have been proposed for classifying cachexia ranging from low BMI to the consensus definition. Patients with COPD often develop other diseases including heart failure and cancer [16] which also can lead to cachexia. Thus, we used the majority of the criteria from the consensus definition [1] to classify cachexia because it was developed by researchers with expertise representative of several diseases including COPD, heart failure and cancer. The consensus definition of cachexia is currently the definition accepted by the Society of Cachexia and Wasting Disorders [17].

We also observed in our Cox proportional hazards models (Table 3) a reduced risk of death for overweight COPD patients in comparison with normal weight COPD patients. A related phenomenon is frequently observed when examining BMI as a cross-sectional measure in diseased populations and has been termed the obesity paradox [18]. However, in our models, which also included the consensus and weight-loss definitions of cachexia in addition to known smoking duration, FEV1pp, sex and age, the reduced risk was only associated with being overweight as opposed to obese. One possible explanation for this observation which we explored was that a magnitude of weight-loss > 5% was not large enough in this population to confer increased risk of death. We ran models examining larger cut points of weight-loss such as weight-loss > 10% and the finding still held (data not shown). Landbo et al. 1999 [19] previously reported reduced risk of death associated with increasing BMI among severe COPD patients and a U-shaped risk profile among those with mild COPD. To assess whether severity of COPD could explain our observation among overweight participants, we contrasted descriptive characteristics between normal and overweight COPD participants (Additional file 4: Table S1) and found all GOLD stages represented among the overweight participants. There were more GOLD 3 and 4 participants in the normal weight group in comparison with the overweight group. For this reason, we adjusted for FEV1% predicted in the Cox Proportional Hazards Models; however, the relationship of reduced risk among the overweight group holds (Table 3). Thus, this observation of reduced risk of death in the Cox Proportional Hazards Models for COPD patients with overweight BMI in comparison with normal BMI is likely the result of unmeasured confounders more prevalent in the overweight population. It is important to note that there was no significant difference in risk of death between the obese BMI group in comparison with the normal weight BMI group in the Cox Proportional Hazards Models. Collectively, the survival models indicate cachexia and weight-loss are associated with increased risk of death independent of BMI group.

Our investigation has several weaknesses. One weakness of the current study is we did not assess whether weight-loss was unintentional. It is possible the observed weight-loss in some individuals was intentional through dieting and/or increased exercise. To alleviate this limitation, we restricted weight-loss coding to those who did not exhibit a re-gain in weight at a later visit over the 3 years of the study. Our logic was based on the unfortunate fact that most diets fail and can be characterized by re-gain of original weight lost [20, 21]. However, we also performed a sensitivity analysis classifying cachexia without removing those who regained weight later in the study and still observed a statistically significant increased risk of death for COPD cases with cachexia (Additional file 1: Figure S1). A further weakness is that we performed a secondary analysis of data primarily collected to understand the etiology of COPD rather than the etiology of cachexia. As a result, some of the criteria used to code the consensus definition of cachexia were not ideal which is why we refer to it as the consensus definition throughout. More specifically, we used low 6MWD as a surrogate of reduced muscle strength whereas the consensus definition recommends the use of handgrip strength [1], which was not measured in the current study. Low 6MWD is not a true measure of muscle strength and more appropriately should be classified as an endurance test. However, 6MWD is commonly administered in epidemiologic studies of COPD whereas strength measures are not. Low 6MWD likely captures COPD cases with extremely reduced muscle strength; however, it may misclassify others. Additionally, all of the criteria to classify abnormal biochemistry (increased CRP or IL6, anemia and low serum albumin) were not available. In general, the bias introduced by misclassification due to incomplete criteria for the consensus definition was most likely towards the null as more COPD cases with weight-loss would have been coded as non-cachectic without the additional information. Further our main results from the survival analyses were consistent whether we defined cachexia using the consensus or weight-loss definition of cachexia. Another weakness is we do not know whether COPD cases in the current study meet the criteria for cachexia due to COPD as opposed to other diseases where cachexia is prevalent such as heart failure and/or cancer. This is one of the reasons that we elected to evaluate cachexia using as much information as possible based on the consensus definition developed by researchers with expertise representative of several diseases including COPD, heart failure and cancer.

## Conclusion

In summary, we report the first study of cachexia using a consensus definition of cachexia among COPD cases. Our investigation revealed the prevalence of cachexia among COPD cases was lower than estimates using only low BMI and low FFMI. Importantly, COPD cases across the entire BMI spectrum were at risk for cachexia which dispels the notion of cachexia being captured exclusively by low BMI. Despite the lower prevalence of cachexia observed using the consensus and weight-loss definitions in the ECLIPSE study, cachectic COPD cases were at a considerably increased risk of death. Monitoring unintentional weight-loss among COPD cases is relatively simple and indicative of patient prognosis. Increased awareness and tracking of weight-loss may facilitate enrolment in clinical trials testing exercise, pharmacological or nutritional intervention in this at-risk population. Future efforts will be directed at recruiting cohorts of COPD cases phenotyped for cachexia in order to further elucidate the etiology of cachexia and expand our understanding of patient prognosis.

## Additional files

Additional file 1: **Figure S1.** Flow-chart depicting number of participants included in analyses. (PDF 22 kb)

Additional file 2: **Figure S2.** Relationship between percent emphysema at baseline and Year 1 with cachexia and weight-loss. (PDF 5 kb)

Additional file 3: **Figure S3.** Relationship between 1-year change in emphysema with cachexia and weight-loss. (PDF 5 kb)

Additional file 4: **Table S1.** Characteristics of ECLIPSE Study COPD cases stratified by BMI category. Continuous variables (age, FEV1pp) are represented by median (IQR). * indicates *P* < 0.05 significant difference from normal BMI category. GOLD was tested as one variable. (DOCX 14 kb)

Additional file 5: **Table S2.** Relationships between risk of death from any cause with consensus and weight loss (WL) definitions of cachexia including COPD cases who regained weight in COPD cases from ECLIPSE. Significant *p*-values are bolded. (DOCX 16 kb)

## Abbreviations

6MWD: 6-Minute Walk Distance
BMI: Body Mass Index
BODE: BMI, Obstruction, Dyspnea and Exercise
CODE: Consensus cachexia, Obstruction, Dyspnea and Exercise
COPD: Chronic Obstructive Pulmonary Disease
CRP: C-reactive protein
ECLIPSE: Evaluation of COPD Longitudinally to Identify Predictive Surrogate Endpoints
FACIT: Functional Assessment of Chronic Illness Therapy
FEV1% predicted: Forced Expiratory Volume in 1 s percent predicted
FFMI: Fat-Free Mass Index
HR: Hazard Ratio
IL6: Interleukin-6
WODE: Weight-loss, Obstruction, Dyspnea and Exercise
