# Supplementary material for: It’s more than low BMI: prevalence of cachexia and associated mortality in COPD
Source: Respir Res. 2019 May 22;20:100. doi: 10.1186/s12931-019-1073-3 (PMC6532157; doi:10.1186/s12931-019-1073-3)
Supplement: Supplementary file 1 — Figure S1. Flow-chart depicting number of participants included in analyses. (PDF 22 kb) [file 12931_2019_1073_MOESM1_ESM.pdf]

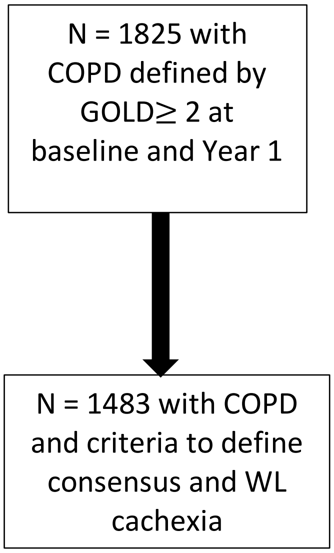
N = 1825 with COPD defined by GOLD≥ 2 at baseline and Year 1
N = 1483 with COPD and criteria to define consensus and WL cachexia
