# Supplementary material for: It’s more than low BMI: prevalence of cachexia and associated mortality in COPD
Source: Respir Res. 2019 May 22;20:100. doi: 10.1186/s12931-019-1073-3 (PMC6532157; doi:10.1186/s12931-019-1073-3)
Supplement: Supplementary file 2 — Figure S2. Relationship between percent emphysema at baseline and Year 1 with cachexia and weight-loss. (PDF 5 kb) [file 12931_2019_1073_MOESM2_ESM.pdf]

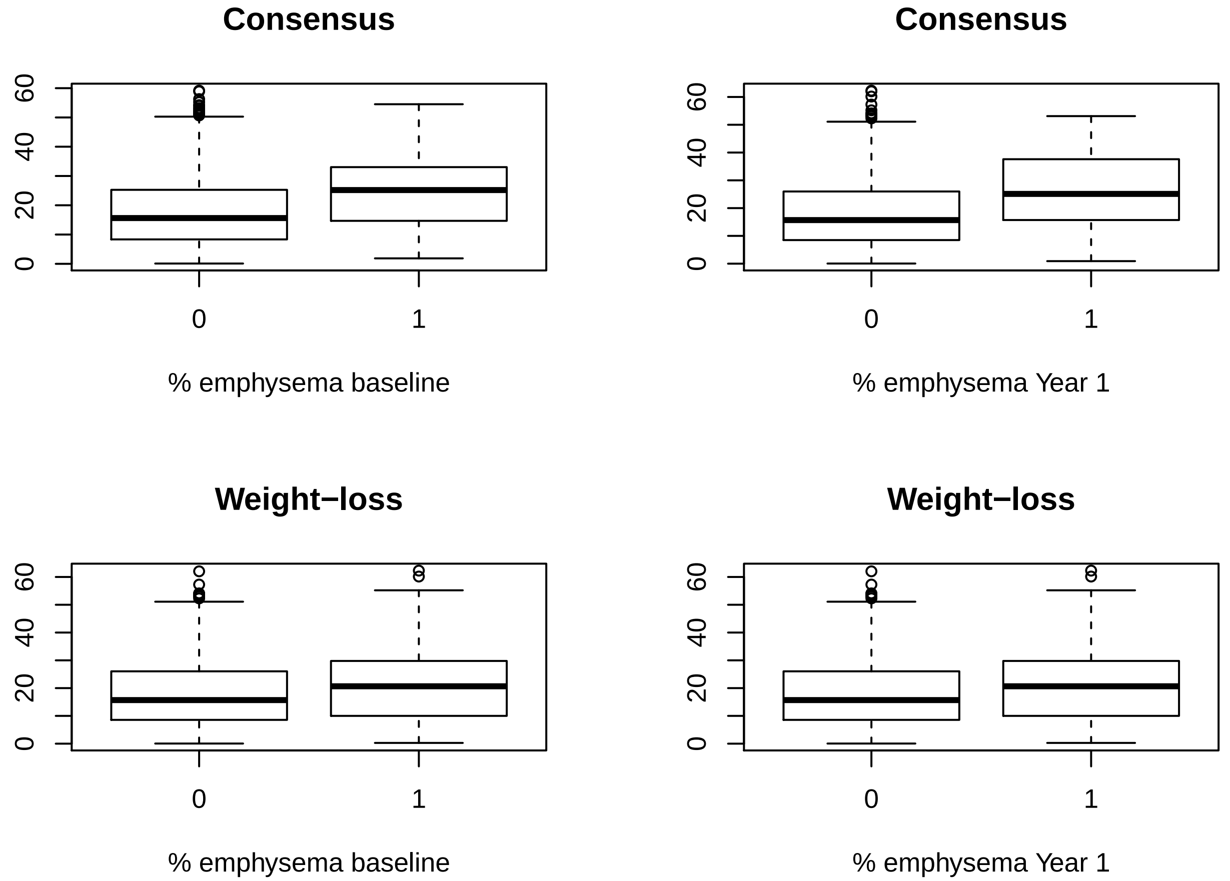
Consensus
% emphysema baseline
Consensus
% emphysema Year 1
Weight−loss
% emphysema baseline
Weight−loss
% emphysema Year 1
0
1
0
20
40
60
