# Supplementary figures and images for: It’s more than low BMI: prevalence of cachexia and associated mortality in COPD
Source: Respir Res. 2019 May 22;20:100. doi: 10.1186/s12931-019-1073-3 (PMC6532157; doi:10.1186/s12931-019-1073-3)

**Consensus**

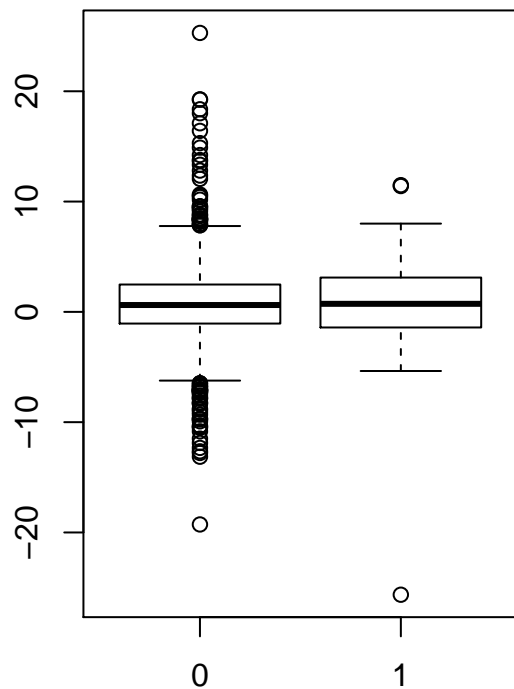

Change in % emphysema

**Weight-loss**

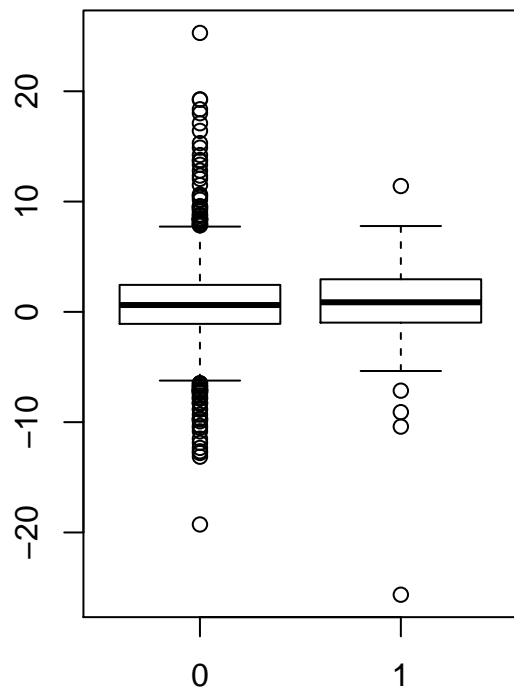

Change in % emphysema

Supplement: Supplementary file 3 — Figure S3. Relationship between 1-year change in emphysema with cachexia and weight-loss. (PDF 5 kb) [file 12931_2019_1073_MOESM3_ESM.pdf]
