# Supplementary material for: It’s more than low BMI: prevalence of cachexia and associated mortality in COPD
Source: Respir Res. 2019 May 22;20:100. doi: 10.1186/s12931-019-1073-3 (PMC6532157; doi:10.1186/s12931-019-1073-3)
Supplement: Supplementary file 4 — Table S1. Characteristics of ECLIPSE Study COPD cases stratified by BMI category. Continuous variables (age, FEV1pp) are represented by median (IQR). * indicates P < 0.05 significant difference from normal BMI category. GOLD was tested as one variable. (DOCX 14 kb) [file 12931_2019_1073_MOESM4_ESM.docx]

| **Supplementary Table 1.** Characteristics of ECLIPSE Study COPD cases stratified by BMI category. Continuous variables (age, FEV1pp) are represented by median (IQR). * indicates P<0.05 significant difference from normal BMI category. GOLD was tested as one variable. | | | | |
| --- | --- | --- | --- | --- |
| **Descriptive** | Low | Normal | Overweight | Obese |
| N | 78 | 559 | 506 | 340 |
| Sex (% Male) | 41* | 64.9 | 68.2 | 67.1 |
| median Age (iqr) | 61 ( 11.8 )* | 64 ( 11 ) | 64 ( 10 ) | 64 ( 9 ) |
| median pack-years (iqr) | 39 ( 29 ) | 40 ( 22 ) | 44 ( 28 ) | 50 ( 31.2 )* |
| median FEV1pp (iqr) | 39.2 ( 17.1 )* | 43.8 ( 24.1 ) | 49.8 ( 23.7 )* | 51.1 ( 21.6 )* |
| GOLD 2 (%) | 20.5 | 38.5 | 48.8 | 52.6 |
| GOLD 3 (%) | 55.1 | 43.3 | 40.9 | 39.1 |
| GOLD 4 (%) | 24.4* | 18.2 | 10.3* | 8.24* |
