# Supplementary material for: It’s more than low BMI: prevalence of cachexia and associated mortality in COPD
Source: Respir Res. 2019 May 22;20:100. doi: 10.1186/s12931-019-1073-3 (PMC6532157; doi:10.1186/s12931-019-1073-3)
Supplement: Supplementary file 5 — Table S2. Relationships between risk of death from any cause with consensus and weight loss (WL) definitions of cachexia including COPD cases who regained weight in COPD cases from ECLIPSE. Significant p-values are bolded. (DOCX 16 kb) [file 12931_2019_1073_MOESM5_ESM.docx]

| **Supplementary Table 2.** Relationships between risk of death from any cause with consensus and weight loss (WL) definitions of cachexia including COPD cases who regained weight in COPD cases from ECLIPSE. Significant p-values are bolded. | | | | |
| --- | --- | --- | --- | --- |
| **Model** | **Covariate** | | **HR (95% CI)** | **P-value** |
| Model 1 | Consensus | | 2.8(1.4-5.7) | **0.004** |
|  | Pack-years | | 1.0 (0.999-1.01) | 0.10 |
|  | BMI category (ref = Normal) | Low | 0.72 (0.25-2.1) | 0.55 |
|  |  | Overweight | 0.29 (0.13-0.64) | **0.002** |
|  |  | Obese | 1.5 (0.86-2.6) | 0.15 |
|  | Sex (ref = males) | | 0.84 (0.48-1.5) | 0.5 |
|  | Age | | 1.1 (1.0-1.1) | **<0.001** |
|  | FEV1 % pred | | 0.98 (96-0.99) | **0.009** |
| Model 2 | Weight-loss | | 2.4 (1.4-4.2) | **0.002** |
|  | Pack-years | | 1.0 (0.999-1.01) | 0.14 |
|  | BMI category (ref = Normal) | Low | 0.73 (0.25-2.1) | 0.56 |
|  |  | Overweight | 0.30 (0.14-0.65) | **0.002** |
|  |  | Obese | 1.5 (0.88-2.6) | 0.13 |
|  | Sex (ref = males) | | 0.80 (0.46-1.4) | 0.44 |
|  | Age | | 1.1 (1.0-1.1) | **<0.001** |
|  | FEV1 % pred | | 0.97 (0.96-0.99) | **0.002** |
| Model 3 | Consensus or weight-loss | | 2.3 (1.3-4.0) | **0.004** |
|  | Pack-years | | 1.0 (0.999-1.01) | 0.13 |
|  | BMI category (ref = Normal) | Low | 0.69 (0.24-2.0) | 0.50 |
|  |  | Overweight | 0.30 (0.14-0.66) | **0.003** |
|  |  | Obese | 1.5 (0.89-2.7) | 0.12 |
|  | Sex (ref = males) | | 0.81 (0.46-1.4) | 0.46 |
|  | Age | | 1.1 (1.0-1.1) | **<0.001** |
|  | FEV1 % pred | | 0.97 (0.96-0.99) | **0.002** |
| FEV1 % pred: forced expiratory volume in 1 second percent predicted, BMI: body mass index | | | | |
